# Phenotypic and Genotypic Detection of Hypervirulent *Klebsiella pneumoniae* Isolated from Hospital-Acquired Infections

**DOI:** 10.3390/microorganisms12122469

**Published:** 2024-12-01

**Authors:** Marwa S. Taha, Reham M. Elkolaly, Mohammed Elhendawy, Hytham Elatrozy, Asmaa Fawzy Amer, Rehab Abd El Fattah Helal, Hanan Salem, Yomna G. El feky, Ahmed Harkan, Raghda Gabr Mashaal, Alzahraa A. Allam, Amira E. Oraiby, Nashwa S. M. Abdeen, Marwa Gamal Bahey

**Affiliations:** 1 Department of Medical Microbiology and Immunology, Faculty of Medicine, Tanta University, Tanta 31527, Egypt; marwabahey@gmail.com; 2 Department of Chest Diseases, Faculty of Medicine, Tanta University, Tanta 31527, Egypt; r.elkolaly@med.tanta.edu.eg; 3 Department of Tropical Medicine and Infectious Diseases, Faculty of Medicine, Tanta University, Tanta 31527, Egypt; elhendawymohamed@gmail.com; 4 Department of Neurosurgery, Faculty of Medicine, Tanta University, Tanta 31527, Egypt; haytham_atrozy@med.tanta.edu.eg; 5 Department of Anesthesia, Surgical Intensive Care, and Pain Medicine, Faculty of Medicine, Tanta University, Tanta 31527, Egypt; asmaafawzyamer@yahoo.com (A.F.A.); rehabhelal2@gmail.com (R.A.E.F.H.); 6 Department of Cardiovascular Medicine, Faculty of Medicine, Tanta University, Tanta 31527, Egypt; hanan.salem@med.tanta.edu.eg; 7 Department of Pediatrics, Faculty of Medicine, Tanta University, Tanta 31527, Egypt; youmna.elfeky@med.tanta.edu.eg (Y.G.E.f.); ahmed.harkan@med.tanta.edu.eg (A.H.); 8 Department of Internal Medicine, Faculty of Medicine, Tanta University, Tanta 31527, Egypt; raghda_mashaal@yahoo.com (R.G.M.); alzahraa.allam@gmail.com (A.A.A.); 9 Department of Clinical Pathology, Faculty of Medicine, Tanta University, Tanta 31527, Egypt; amira.oreiby@gmail.com (A.E.O.); nashwa.sallam@med.tanta.edu.eg (N.S.M.A.)

**Keywords:** hypervirulent, *Klebsiella pneumoniae*, aerobactin-positive, *rmpA* gene, *iuc A* gene, capsular serotypes, hospital-acquired infections

## Abstract

Hypervirulent *Klebsiella pneumoniae* is a highly pathogenic variant of *Klebsiella pneumonae*, which represents a global public health issue because it is very virulent and spreads easily. The objectives of this study were to assess the predominance of hvKp among health care-associated infections in intensive care units of Tanta University Hospital and to compare hvKp with classical *K. pneumoniae* (cKp) in terms of antibiotic resistance, virulence, and molecular features. The study included 300 patients suffering from HAIs from different ICUs of Tanta University Hospitals. *K. pneumoniae* isolates were identified and subjected to string testing and antibiotic susceptibility testing, and the tissue culture assay for biofilm formation and polymerase chain reaction (PCR) tests were performed for the identification of capsular genes (*K1*, *K2*, *K57*) and virulence genes (*rmpA*, *rmpA2*, *iuc A*). Fifty-seven *K. pneumonaie* isolates were isolated. A total of 21 (36.8%) of them were hvKp and 36 (63.15%) were cKp. Significantly higher antibiotic resistance was detected in the cKp group. There was a significant difference between biofilm formation between cKp and hvKp isolates (*p* < 0.004*). *iucA*, *rmpA2*, and *K1* genes were significantly associated with hvKp. The string test shows 100% sensitivity and negative predictive value for the detection of hvKp. Consequently, using the string test alone for the screening of hvKp is required. However, combining aerobactin-positive with hypermucoviscous isolates while screening for hvKp is crucial.

## 1. Introduction

Hypervirulent *Klebsiella pneumoniae* (hvKp) is a Gram-negative bacterium that is encapsulated, nonmotile, and does not produce spores. It is a highly virulent strain of *K. pneumoniae* that was first identified in Taiwan during the mid-1980s [1]. High percentages of hospital-acquired infections (HAIs) and severe community-acquired infections in Egypt are caused by *K. pneumoniae* isolates [2].

Nonvirulent, classical *K. pneumoniae* infections can lead to pneumonia, urinary tract, wound, and blood infections. Nevertheless, in immunocompromised and young healthy individuals, hvKp isolates produce many more serious illnesses like pyogenic liver abscesses, meningitis, urinary tract infections, necrotizing ascites, and endophthalmitis [3].

The production of biofilm is much higher in hvKp strains compared to cKp strains, suggesting a potential role for biofilm development in hvKp pathogenesis [4]. Moreover, hvKp infections are frequently linked with deadly disseminated infections and have a mortality rate varying from 3 to 42% [5].

Some strains of hvKp that are resistant to tigecycline, polymyxin, carbapenemase, and extended-spectrum β-lactamases (ESBLs) have now steadily emerged [6]. The global spread of the mobile genetic components, which carry many antibiotic-resistance genes, including *oxacillinases-48 (OXA-48*), *New Delhi metallo-beta-lactamase 1 (NDM)*, and *carbapenemases* from *K. pneumoniae*, has led to the evolution of antibiotic-resistant hvKp strains [7,8].

Hypermucoviscous *K. pneumonia* (hmvKp) isolates have a high pathogenicity level, according to many investigations that used in vitro and in vivo tests [9,10]. Throughout the course of the *K. pneumoniae* infection process, the capsule is essential, which plays a role in mucoviscosity. A straightforward string test on a bacterial colony plate reveals that most hvKp strains are quite viscous. This test may be performed to confirm their identity. Hypermucoviscosity is a characteristic, non-specific phenotypic feature of the hvKp strain [11].

Researchers have identified significant virulence genes that are more reliable for identifying hypervirulence because of advancements in genomic analysis. Aerobactin is thought to be an important virulence factor for hvKp, which is frequently linked with the hypermucoviscous phenotype [12]. This resulted in studies that were carried out across several centers in China which initially reported the clinical and molecular features of hvKp (classified as aerobactin-positive) isolates [13].

The virulence-associated genes have been studied as potential molecular markers for hvKp identification [14]. The most well-known virulence factors that have experimental evidence for identifying the hypervirulent phenotype are *iucA* (biosynthetic genes for such siderophore aerobactin), *rmpA*, *rmpA2* (regulators that encourage the development of capsules), and *Peg-344* (metabolic transporter) [12,15]. These hypervirulence-related features of hvKp can be exploited to design novel therapies, because hvKp infection management is difficult [5].

The role of hvKp in community-acquired infections has been shown in several research areas, while its role in HAI has not been well-studied [16,17]. Consequently, the objective of this study was to evaluate the frequency of hvKp among patients with HAIs in intensive care units (ICUs) of Tanta University Hospitals and to compare hvKp with cKp in terms of antibiotic resistance, virulence, and molecular features.

## 2. Materials and Methods

### 2.1. Study Design

Over the period of six months, from January 2024 to June 2024, this cross-sectional study was conducted in the Medical Microbiology, Immunology, and Clinical Pathology Departments at Tanta University Hospitals. The hospitals serve more than 190,000 patients a year and have a total capacity of 2040 beds, including 130 ICU beds. The Institutional Review Board of Tanta University granted authorization for the study to be conducted in the Faculty of Medicine in Egypt. (Reference number: 36264PR588/3/24).

### 2.2. Study Subjects

In this study, 300 ICU patients from Chest, Pediatric, Internal Medicine, Cardiology, and Anesthesia Departments of Tanta University hospitals were enrolled. The diagnosis for the included individuals was hospital-acquired infections. Within 48 h of admission, patients with a clinically verified infection were not included. Clinical isolates of *Klebsiella* were examined, originating from various fluids in the body, such as the blood, urine, wounds, and sputum; the bronchoalveolar lavage method was also used. The same patient’s duplicate isolates were not included.

### 2.3. Bacterial Isolate Identification

Different samples were collected and expeditiously forwarded to the Microbiology Department’s laboratory for further analysis. After being codified, the samples were grown aerobically for 24 to 48 h at 37 °C on blood agar, nutrition agar, chocolate agar, MacConkey agar, and CLED agar plates (Oxoid, UK). For the phenotypic identification of isolated pathogens, standard microbiological techniques were primarily employed [18]. Only *K. pneumonia* isolates were processed further after that. The isolates were confirmed in compliance with manufacturer guidelines using the Vitek-2 automated system (Biomérieux, Marcy-LÉtoile, France). The string test verified the definition of the hvKp strains as hypermucoviscous. Until they were required, all *K. pneumoniae* isolates were stored at −80 °C in brain heart infusion broth (BHI) (20% glycerol; Oxoid, UK).

### 2.4. Antimicrobial Susceptibility Testing

On Muller–Hinton agar (Oxoid, UK) plates, the antibiotic susceptibility of every *K. pneumoniae* isolate found was evaluated using the Kirby Bauer disc diffusion technique with modifications. A direct bacterial colony suspension equivalent to a 0.5 McFarland standard was made from a fresh pure 24 h culture to prepare the inoculum. The antibiotics used were 5 μg of ciprofloxacin (CIP), 30 μg of cefuroxime (CXM), 110 μg of piperacillin–tazobactum (TPZ), 30 μg of cefoxitin (FOX), 30 μg of cefipime (FEP), 30 μg of ceftriaxone (CRO), 30 μg of ceftazidime (CAZ), 30 μg of cefotaxime (CTX), and 10 μg of imipenem (IMI). Additionally, 10 μg of ertapenem (ERT) and 10 μg meropenem (MEM) (Oxoid, UK) were used. *Klebsiella pneumoniae* ATCC BAA-1705 was utilized as a positive control and *E. Coli* ATCC 25922 was used as a susceptible strain. The CLSI 2023 criteria were used to evaluate the data produced by the susceptibility assay [19].

### 2.5. String Test

The bacterial colonies that were cultivated overnight on a blood agar plate at a temperature of 37 degrees Celsius were elongated using a typical bacteriological loop. A mucoviscous string longer than 5 mm was deemed a positive result for the string test, indicating that the isolate has a high mucoviscosity [20].

### 2.6. Biofilm Development

The production of biofilm was assessed using a semi-quantitative test in 96-well flat bottom plates, as described in a previous study [21]. Each test organism was grown on a 96-well plate using a trypticase soy broth (TSB) medium and incubated for 24 h. After three rounds of washing in sterile phosphate buffered saline (PBS) to get rid of the free-floating bacteria, the plates were dried. To stain biofilms, 200 μL of crystal violet (0.1%) was applied and left for 15 min. The biofilms were rinsed three times with saline (PBS) to eliminate free-floating bacteria before being dried. Bacterial attachments became soluble with 200 μL of 95% ethanol, and the opacity was monitored at OD570 using an ELISA reader. The biofilm development was calculated in comparison to the control’s mean OD (uninoculated TSB). There were four groups of isolates: non-biofilm producers (ODT <  ODC), weak-biofilm producers (ODC < ODT < 2 × ODC), moderate-biofilm producers (2 × ODC < ODT < 4 × ODC), and strong-biofilm producers (4 × OD C < ODT).

### 2.7. PCR for Recognition of Genes Associated with Virulence and Capsular Serotype

The polymerase chain reaction (PCR) was used to detect genes related to capsular serotypes (*K1*, *K2*, *K5*, and *K57*) and virulence (*rmpA*, *rmpA2*, and *iuc A*). The *iuc A* gene was utilized for the identification of hvKp. The DNA extraction of all individual *K. pneumoniae* strains was performed using the boiling procedure, as previously outlined [22]. The polymerase chain reaction (PCR) method utilized for the amplification of genes specific to capsular serotypes (*K1*, *K2*, *K5* and *K57*) was performed according to the method reported previously [17]. The PCR method was used to determine the presence of virulence-associated genes (*rmpA* and *rmpA2*), following the procedure outlined in earlier studies [23,24]. To amplify *iucA* (aerobactin), the following PCR conditions were used: 15 min of initial denaturation at 95 °C, followed by denaturation at 95 °C for 15 s, annealing at 49 °C for 15 min, and extension at 72 °C for 1 min. This cycle was repeated 30 times, and a final extension step was performed at 72 °C for 10 min. The primers utilized in this investigation are enumerated in Table 1. Next, the PCR products underwent electrophoresis at a voltage of 100 V for a duration of 2 h in a gel made of 2% agarose and containing ethidium bromide at a concentration of 0.5 g/mL. The DNA bands were detected using UV light at a wavelength of 302 nm on a UV transilluminator.

### 2.8. Statistical Analysis

SPSS version 20 (SPSS Inc., Chicago, IL, USA) is an analysis program for the social sciences that is used to tabulate and analyze data. When comparing groups, either the Fisher’s exact test or the Chi-square test was applied. Significant results were those with a *p*-value of less than 0.05. Sensitivity, specificity, positive predictive value, negative predictive value, and total accuracy were used to describe the diagnostic effectiveness of the string test.

## 3. Results

### 3.1. Patient Characteristics

Out of 300 non-duplicate samples taken from different hospital-acquired infections, 57 *K. pneumonia* isolates were detected. Considering the string test’s findings, 21 (36.8%) of these isolates resulted in a positive string test and were designated as hmvKp. The remaining 36 isolates (63.15%) were string test-negative and were designated as cKp. Eventually, it was shown that only 18 of the 21 isolates were hypervirulent following the PCR identification of virulence genes.

Males were more frequent affected than females both in hvKp and cKp, with percentages of 71.4% and 80.6%, respectively. The mean age ± SD of patients with hvKp infections was 49.57 ± 11.66years. The mean age ± SD of patients with ckp was 50.06 ± 11.44 years. The age range was 43:60 in hvKp and nearly similar in cKp (44:58). Neither age nor sex were significantly associated with hvKp.

The highest number of hvKp isolates were from the chest ICU and the highest number of cKp isolates were from the internal medicine ICU, with 42.9% and 36.1%, respectively. Both hvKp and cKp infections were more prevalent in patients with pulmonary disease, followed by patients with diabetes mellitus (DM); as a percentage, 42.9% were in hvKp infections vs. 27.8% in cKp infections for pulmonary disease, and 33.3% in hvKp infections vs. 19.4% in cKp infections for DM. No significant differences in the underlying conditions of patients were recorded between hvKp and cKp strains.

Regarding the type of infection, hvKp infections were isolated as follows: nine isolates (42.9%) were from ventilator-associated pneumonia (VAP), five isolates (23.8%) were from urinary tract infections (UTIs), five isolates (23.8%) were from septicemia, and two (9.5%) were isolated from surgical site infections (SSIs). The cKp infections were isolated from UTIs, VAP, SSIs, and septicemia, with percentages of 30.6%, 27.8%, 25%, and 16.7%, respectively. Patients with hvKp and cKp infections are displayed together with their clinical and demographic characteristics in Table 2.

### 3.2. Antibiotic Resistance Pattern in All K. pneumonia Isolates

As for resistance phenotypes based on the disc diffusion method, both hvKp and cKp exhibited a high antimicrobial resistance rate for all tested antibiotics except tigecycline, which showed the lowest resistance rate at 9.5% and 36.1% in hvKp and cKp, respectively. However, cKp showed a significantly higher resistance rate for all tested antibiotics than hvKp isolates, with a statistically significant difference (*p* ≤ 0.05). We detected a significant difference in cefoxitin, ceftriaxone, cefepime, aztreonam, ciprofloxacin, levofloxacin, and tigecycline with *p*-values 0.026, 0.001, 0.005, 0.003, 0.048, 0.048, and 0.028 respectively. For hvKp, the highest resistance rate was for piperacillin tazobactam, ceftazidime, and meropenem, with a percentage of 47.6% for all. For cKp, the highest resistance rate was for ceftriaxone at 80.6% Multidrug-resistant isolates were represented in 38.1% and 63.9% of hvKp and cKp infections, respectively, with non-significant differences (Table 3).

### 3.3. Comparing hvKp and cKp Isolates in Relation to String Test and Biofilm Formation

Depending on the string test, the hypermucoviscous phenotype was detected in 21 out of 57 isolates of all k. pneumoniae cases. The string test was positive in all hvKp isolates and negative in all cKp isolates (100% vs. 0%), with a statistically significant difference (*p* < 0.001*). Upon measuring the optical density (OD) of the 96-well flat bottom plates using an ELISA reader, high levels of biofilm development were more strongly linked to the hvKp isolates (76.2%) than the cKp isolates (44.4%). However, non-biofilm formation was more strongly linked to cKp isolates (38.9%) than hvKp isolates (0%), with a significant difference *p* = 0.004*, as represented in Table 4.

### 3.4. Molecular Detection of Different Virulence Genes and Serotypes in hvKp and cKp

Virulence gene detection was performed using PCR with agarose gelelectrophoresis, which separated bands according to their size and charge, and DNA bands were detected using UV light. Our results revealed that *rmpA*, *rmpA2*, and *iucA* genes were present more frequently in hvKp isolates than cKp isolates, with variable percentages of 28.6%, 81%, and 100%, respectively. The positive rate of *rmpA2* and *iucA* genes was significantly higher in hvKp isolates than cKp isolates (*p*-value < 0.001*). The *rmpA* gene was present in a higher percentage in hvKp isolates than cKp isolates, without a significant difference (*p*-value = 0.327). Furthermore, capsular serotype-specific genes (*K1*, *K2*, and *K57*) were present more frequently in hvKp isolates than cKp isolates, with percentages of 47.6%, 23.8%, and 4.8%, respectively, with a significant difference for *K1* gene *p* = 0.002*. However, most cKp isolates (77.8%) were non-typable, with a significant difference (*p* < 0.001*). Data are represented in Table 5.

The hvKp isolate was confirmed by the presence of *iucA* genes and one of the following two genes: *rmpA* or *rmpA2.* The study showed that two out of twenty-one positive string test (hypermucoviscous) isolates were positive for *iuc A* and negative for both *rmpA* and *rmp A2*. Also, three isolates of hvKp were positive for all virulence genes: *iucA*, *rmpA*, and rmpA2. The phenotypic and genotypic characteristics of all hvKp isolates are presented in Table 6.

Given that the PCR method is the gold standard, the accuracy, sensitivity, specificity, and positive and negative predictive values of the string test for the recognition of hvKp isolates were 100%, 94.74%, 90.84, 100%, and 96.49%, respectively, as shown in Table 7.

## 4. Discussion

Metastatic and potentially fatal infections in immunocompetent healthy young people are clinically defined by the variance in hvKp, a pathotype of *K. pneumoniae* that is becoming more and more documented [25]. To account for geographical disparities in hvKp infection, this study examined the prevalence, risk variables, and molecular features of hvKp in our tertiary care facilities to enhance the comprehension of its threats.

The present study detected 57 *K. pneumoniae* isolates that were isolated from different HAI samples. According to the string test results, 21 isolates (36.8%) were positive and classified as hvKp. A total of 36 (63.15%) of the remaining isolates were string test-negative and were classified as ckp. However, it was shown that only 18 of the 21 isolates were hypervirulent following the PCR identification of virulent genes. Our findings are consistent with other research projects in Egypt, which found the hypermucoviscous phenotype in approximately 40% of *K. pneumoniae* isolates [26]. Another finding from an additional study established in Sohag, Egypt, reported that the string test was positive in 28% of hvKp cases [27]. However, our percentage was higher than that reported by Emam et al. [28], who found that hmvKp was demonstrated by a positive string test in 17.1% (12/70) of the *K. pneumoniae* isolates. Furthermore, El-Mahdy et al. [2], found that 13.8% of *K. pneumoniae* isolates in Mansoura, Egypt, were hmvKp, whereas 86.2% were cKp. The variation in sample size can explain this mismatch.

We determined that the highest number of hvKp isolates were from the chest ICU, while the highest number of cKp isolates were from the internal medicine ICU, with percentages of 42.9% and 36.1%, respectively. These results are in accordance with Mohamed et al. [27], who reported that hvKp and cKp were mostly found in the intensive care unit. Furthermore, according to other research, an increasing number of hvKp strains were discovered in chest and pediatrics ICUs [29]. Variations in rates may be due to factors such as regional dispersion, patient immunity, and hospital infection control efforts.

Our results showed that males were more frequently affected than females both in hvKp and cKp, with percentages of 71.4% and 80.6%, respectively. According to Mohammed et al. [27], males outnumber females in both hvKp and cKp (42% vs. 26%). Additionally, another study held in Saudi Arabia reported that 55% patients were males and 45% were females [25].

Our results revealed that the mean age ± SD of patients with hvKp infections was 49.57 ± 11.66 years, while the mean age ± SD of patients with ckp was 50.06 ± 11.44 years. The age range was 43: 60 in hvKp infections and similar in cKp infections (44:58). There was no significant correlation between hvKp infection and either sex or age. This was in accordance with [27], who found that the patients with hvKp infections had a mean age ± SD of 62.7 ± 20.5 years and 63.2 ± 24.9 in ckp infections, and the range for age was 48:82 years. This was similar to results obtained by the authors of [30], who observed a mean age ± SD of 57 ± 14; however, these results are different from those in [31], in which it was claimed that hvKp infection was common in young individuals.

Regarding the underlying disease, we reported that both hvKp and cKp isolates were more prevalent in patients with pulmonary disease, followed by patients with diabetes mellitus. This agreed with Ding et al. [31], who found that hvKp infection was more common in diabetic patients. Moreover, Emam et al. [28] stated that there was a significant correlation between DM and the development of hvKp, since it was seen in 75% of isolates. Conversely, other research has shown that hvKp commonly causes invasive infections in young individuals who do not have any underlying medical conditions [32,33]. But these findings were contrary to ours.

Despite hvKp being recognized as a prevalent blood isolate [34,35], few research projects have identified it in alternative samples [36]. Our analysis indicated that the majority of hvKp isolates (42.9%) were isolated from VAP samples, while the fewest isolates were identified from SSI samples. Comparable results were achieved by other researchers [26,36]. Conversely, Li et al. [32] found that hospital-acquired hvKp was more common in ascites and blood samples.

Concerning antimicrobial resistance, our results showed that both hvKp and cKp exhibited a high antimicrobial resistance rate for all tested antibiotics except tigecycline, which showed the lowest rate at 9.5% and 36.1% in hvKp and cKp, respectively. But cKp isolates showed a significantly higher resistance rate for all tested antibiotics than hvKp isolates, with a statistically significant difference. This aligned with other studies conducted in Egypt and China, which revealed identical outcomes [25,37]. This may be explained by another study, which reported that compared to cKp, hvKp had a more difficult time acquiring plasmids encoding antibiotic resistance. This finding was supported by the population genomics data. A possible explanation proposed by the authors is that the capsule is over-expressed, creating a physical barrier that prevents the transfer of antimicrobial resistance genes [38]. Nevertheless, a separate study carried out in Egypt indicated that there was no substantial disparity in the antimicrobial resistance patterns between hvKp and cKp strains [2].

Our findings detected a high percentage of multidrug resistance and even carbapenem resistance in hvKp isolates, which disagreed with the previous study results of Abd-Elmonsef et al. [26], who found that lowest resistance rates were for carbapenems, as none of the hmvKp isolates were resistant to them and only 2.99% (2/67) of cKP isolates were resistant to imipenem. This discrepancy may be explained by the fact that our patients used this antibiotic more often.

The hvKp isolates detected in the present study were much more closely linked to biofilm development (76.2%) than cKp isolates (44.4%). The cKp isolates were more highly associated with non-biofilm formation (38.9%) than hvKp isolates (0%), with a significant difference. This is consistent with the results of a previous study that reported the same data [39]. Another study in Egypt found no significant difference between hvKp and cKp in terms of biofilm formation [2]. The development of biofilm gives *K. pneumoniae* species better defense against host immunological responses, antibiotic action, and increases its persistence. [13].

With reference to the virulence factors, in *K. pneumoniae*, the capsule is thought to be the primary virulence factor; there are various kinds of K-antigens [40]. According to the literature, the most significant serotypes are *K1* and *K2*, as they often cause serious illnesses [41]. Our results showed that hvKp is more likely to include specific genes for capsular serotypes (*K1*, *K2*, and *K57*) than cKp. Alharbi et al. [25] reported identical outcomes. Furthermore, *K1*, *K2*, *K3*, *K5*, and *K20* accounted for 46.7% of the *K. pneumoniae* isolates, according to Ssekatawa et al. [42]. Additionally, our findings align with research by Fung et al. and Chuang et al. [43,44], which determined that the most virulent capsular types of *K. pneumoniae*, *K1* and *K2*, were implicated in septicemia and liver abscess. Nevertheless, Taha et al. [45] stated that *K1* is the most capsular gene carried by *K. pneumoniae* isolates, whereas *K2* is the least capsular gene.

A combination of phenotypic and genotypic traits can be utilized to distinguish between the hvKp and cKp strains embracing the mucoid phenotype regulator A *(rmpA* and *rmpA2*). Furthermore, aerobactin (*iucA)* was a key virulence factor for the increased siderophore synthesis in hvKp strains and was utilized to identify hvKp [46].

Our study found a substantial association between aerobactin *(iucA*) and hvKp, with a statistically significant difference, as indicated by Alharbi et al. [25].

Moreover, the *rmpA* and *rmpA2* genes, which produce the hypermucoviscous phenotypes, are regarded as additional virulence determinants [47,48]. We determined that hvKp had higher levels of the *rmpA*, *rmpA2*, and *iucA* genes than cKp, with a variable percentage. Also, the positive rate of *rmpA2* and *iucA* genes were significantly higher in hvKp isolates than cKp isolates (*p*-value < 0.001*). The *rmpA* gene was present in a higher percentage in hvKp than cKp, without asignificant difference. These findings are in line with a prior investigation that found a strong correlation between these genes and hvKp but not cKp [37]. In contrast to this, additional findings indicated that *rmpA* and *rmpA2* were not substantially correlated with hvKp strains [25,31].

According to Russo et al. [49], hvKp can be identified by the presence of *iucA* with one or both *rmpA* and *rmpA2* genes using the PCR method. Our results revealed that out of twenty-one string test-positive isolates, two isolates were positive only for *iucA* and negative for *rmpA* and *rmpA2.* Therefore, these isolates were hypermucoviscous, not hypervirulent. Also, *rmpA* and *rmpA2* genes were represented by 28.6% and 81% of hvKp isolates in our study, respectively, which is lower than the results of El-Mahdy et al. [2] in Egypt.

In the current investigation, the PCR method was used as the gold standard to evaluate the string test’s performance and accuracy as a simple approach for hvKp laboratory diagnosis. The test had 100% sensitivity, 94.74% specificity, 90.84% positive predictive value (PPV), 100% negative predictive value (NPV), and 96.49% accuracy. Our findings concurred with those of prior research by Russo and Gulick, [49] who found 90% accuracy, 89% specificity, and 91% sensitivity for the string test. Furthermore, Tan et al. [50] discovered that the string test had a 97.2% NPV, 32.7% PPV, 90.5% sensitivity, and 63.9% specificity. Nevertheless, other studies revealed that not all hvKp strains had a hypermucus phenotype. Rather, an increasing number of cKp strains exhibited the hypermucus phenotype. Due to its ease of use and simplicity, it could be used as an NPV test [51].

## 5. Conclusions

Our study demonstrates how common hvKp is in our hospital, mainly affecting ICU patients with hospital-acquired infections. Due to its resistance profiles, which are alarming and rapidly changeable, and its biofilm-forming ability, infection control protocols must be strictly implemented. HvKp strains were not related to specific serotypes. The string test is an easy-to-use test as a preliminary test to screen for the presence of hvKp in any lab. However, both serotypes and the aerobactin genetic background may provide confirmatory methods for detection.

## Figures and Tables

**Table 1:** Primer sequences used in molecular detection of capsular serotype-specific genes and virulence-associated genes of *K. pneumonia*.

Primers Targeting Capsular-Encoding Genes			
Target Genes	Primer Sequence	Amplicon Size (Bp)	Reference
*rmpA*	F: 5′-ACTGGGCTACCTCTGCTTCA-3′ R: 5′-CTTGCATGAGCCATCTTTCA-3′	428	[23]
*rmpA2*	F: 5′-CTTTATGTGCAATAAG-GATGTT-3′ R: 5′-CCTCCTGGAGAGTAAGCATT-3′	1283	[24]
*iuc A*	F: 5′-GCATAGGCGGATACGAACAT-3′ R: 5′-CACAGGGCAATTGCTTACCT-3	641	[10]
*K1*	F: 5′-GTAGGTATTGCAAGCCATGC-3′ R: 5′-GCCCAGGTTAATGAATCCGT-3′	280	[17]
*K2*	F: 5′-GGAGCCATTTGAATTCGGTG-3′ R: 5′-TCCCTAGCACTGGCTTAAGT-3′	741	[17]
*K5*	F: 5′-GCCACCTCTAAGCATATAGC-3′ R: 5′-CGCACCAGTAATTCCAACAG-3′	881	[17]
*K57*	F: 5′-CGACAAATCTCTCCTGACGA-3′ R: 5′-CGCGACAAACATAACACTCG-3′	1037	[17]

BP: Base pair: F: Forward: R: Reverse.

**Table 2:** Demographic and clinical characteristics of patients hypervirulent *Klebsiella pneumoniae* (hvKp) and classical *Klebsiella pneumoniae* (cKp) isolates.

	hVKp (n = 21)	CKp (n = 36)	Test of Sig.	*p*-Value
Sex				
Male	15 (71.4%)	29 (80.6%)	χ^2^ = 0.628	^FE^*p* = 0.58
Female	6 (28.6%)	7 (19.4%)		
Age (years)				
Min.–Max.	6–67	9–73	t = 0.153	0.879
Mean ± SD.	49.57 ± 11.66	50.06 ± 11.44		
Median (IQR)	50(43–60)	49(44–58)		
ICU type				
Int. med	6 (28.6%)	13 (36.1%)	FET = 3.163	0.556
Anaesth	1 (4.8%)	5 (13.9%)		
Pediatric	4 (19%)	4 (11.1%)		
Chest	9 (42.9%)	10 (27.8%)		
Cardiac	1 (4.8%)	4 (11.1%)		
Comorbidity				
Non	0 (0%)	1 (2.8%)	FET = 6.533	0.487
Cancer	1 (4.8%)	3 (8.3%)		
Cardiovascular disease	2 (9.5%)	5 (13.9%)		
Pulmonary disease	9 (42.9%)	10 (27.8%)		
Renal failure	0 (0%)	2 (5.6%)		
Diabetes mellitus (DM)	7 (33.3%)	7 (19.4%)		
Hypetension (HTN)	0 (0%)	5 (13.9%)		
DM+ HTN	2 (9.5%)	3 (8.3%)		
Type of infection				
SSI	2 (9.5%)	9 (25%)	FET = 3.066	0.398
UTI	5 (23.8%)	11 (30.6%)		
VAP	9 (42.9%)	10 (27.8%)		
Septicemia	5 (23.8%)	6 (16.7%)		

SD: Standard deviation; t: Student *t*-test; c^2^: Chi-square test; FET: Fisher’s exact test; *p*: *p*-value for comparing between the studied groups.

**Table 3:** Antimicrobial resistance pattern of hypervirulent *Klebsiella pneumoniae* (hvKp) and classical *Klebsiella pneumoniae* (cKp) isolates.

Antibiotics	Abbreviations Disk Content (μg)	hvKp (n = 21)		CKp (n = 36)		χ^2^	*p*-Value
		N	%	N	%		
PipracillinTazobactam	TZP 100/10	10	47.6%	21	58.3%	0.614	0.433
Cefoxitin	FOX 30	7	33.3%	23	63.9%	4.967 *	0.026 *
Ceftriaxone	CRO 30	8	38.1%	29	80.6%	10.499 *	0.001 *
Ceftazidime	CZC 30	10	47.6%	26	72.2%	3.450	0.063
Cefipime	FEP 30	6	28.6%	24	66.7%	7.721 *	0.005 *
Aztreonam	ATM 30	8	38.1%	28	77.8%	8.976 *	0.003 *
Imipenem	IPM 10	9	42.9%	20	55.6%	0.856	0.355
Meropenem	MEM 10	10	47.6%	21	58.3%	0.614	0.433
Gentamycin	CN 10	8	38.1%	23	63.9%	3.557	0.059
Ciprofloxacin	CIP 5	9	42.9%	25	69.4%	3.895 *	0.048 *
Levofloxacin	LEV 5	9	42.9%	25	69.4%	3.895 *	0.048 *
Tigycycline	TGC 30	2	9.5%	13	36.1%	4.835 *	0.028 *
Multi Drug Resistant (MDR)		8	38.1%	23	63.9%	3.557	0.059

χ^2^: Chi-square test; *p*: *p*-value for comparing between the studied groups; *: Statistically significant at *p* ≤ 0.05.

**Table 4:** Comparison between hypervirulent *Klebsiella pneumoniae* (hvKp) and classical *Klebsiella pneumoniae* (cKp) regarding string test and biofilm formation.

	hvKp (n = 21)	cKp (n = 36)	χ^2^	*p*-Value
String test				
Negative	0 (0%)	36 (100%)	57.00 *	<0.001 *
Positive	21 (100%)	0(0%)		
Biofilm formation				
Non (ODT < ODC)	0 (0%)	14 (38.9%)	10.898 *	0.004 *
Moderate (2 × ODC < ODT < 4 × ODC)	5 (23.8%)	6 (16.7%)		
Strong (4 × ODC < ODT)	16 (76.2%)	16 (44.4%)		

χ^2^: Chi-square test; *: Statistically significant at *p* ≤ 0.05. ODT: Optical density of test well, ODC: Optical density of control well.

**Table 5:** Distribution of virulence genes and serotypes in hvKp and cKp.

	hvKp (n = 21)	cKp (n = 36)	χ^2^	*p*-Value
*rmpA*				
Negative	15 (71.4%)	30 (83.3%)	1.131	0.327
Positive	6 (28.6%)	6 (16.7%)		
*rmpA2*				
Negative	4 (19%)	33 (91.7%)	30.709 *	<0.001 *
Positive	17 (81%)	3 (8.3%)		
*iuc A*				
Negative	0 (0%)	36 (100%)	57.0 *	<0.001 *
Positive	21 (100%)	0 (0%)		
Serotypes				
Non typable	5 (23.8%)	28 (77.8%)	15.847	<0.001 *
*K1*	10 (47.6%)	3 (8.3%)	11.627	^FE^*p* = 0.002 *
*K2*	5 (23.8%)	3 (8.3%)	2.633	^FE^*p* = 0.130
*K57*	1 (4.8%)	2 (5.6%)	0.017	^FE^*p* = 1.000

χ^2^: Chi-square test; FET: Fisher’s exact test; *: Statistically significant at *p* ≤ 0.05.

**Table 6:** Phenotypic and genotypic characteristics of different hypervirulent *Klebsiella pneumoniae* isolates.

Sample n.	*rmpA*	*rmpA2*	*iuc A*	Serotypes	String Test	Biofilm
hv *Kp 1*	Positive	Positive	Positive	Non	Positive	Strong
hv *Kp 2*	Positive	Positive	Positive	Non	Positive	Strong
hv *Kp 3*	Positive	Positive	Positive	Non	Positive	Strong
hv *Kp 4*	Negative	Positive	Positive	Non	Positive	Moderate
hv *Kp 5*	Negative	Positive	Positive	*K57*	Positive	Moderate
hv Kp 6	Negative	Positive	Positive	*K1*	Positive	Strong
hv *Kp 7*	Negative	Positive	Positive	*K1*	Positive	Strong
hv *Kp 8*	Negative	Positive	Positive	*K1*	Positive	Strong
hv *Kp 9*	Negative	Positive	Positive	*K1*	Positive	Strong
hv *Kp 10*	Negative	Positive	Positive	*K1*	Positive	Strong
hv *Kp 11*	Negative	Positive	Positive	*K1*	Positive	Strong
hv *Kp 12*	Negative	Positive	Positive	*K1*	Positive	Strong
hv *Kp 13*	Negative	Positive	Positive	*K2*	Positive	Strong
hv *Kp 14*	Negative	Positive	Positive	Non	Positive	Moderate
hv *Kp 15*	Negative	Positive	Positive	*K2*	Positive	Strong
hv *Kp 16*	Negative	Positive	positive	*K2*	Positive	Strong
hmv *Kp 17*	Negative	Negative	Positive	*K1*	Positive	Moderate
hmv *Kp 18*	Negative	Negative	positive	*K1*	Positive	Moderate
hv *Kp 19*	Positive	Positive	Positive	*K2*	Positive	Strong
hv *Kp 20*	Positive	Negative	Positive	*K2*	Positive	Strong
hv *Kp 21*	Positive	Negative	Positive	*K1*	Positive	Strong

**Table 7:** Sensitivity, specificity, positive and negative predictive values, and accuracy of string test (n = 57).

	Gold Standard (PCR)		Sensitivity	Specificity	PPV	NPV	Accuracy
	Negative (n = 38)	Positive (n = 19)					
String test							
Negative	36 (94.7%)	0 (0)	100	94.74	90.48	100	96.49
Positive	2 (5.3%)	19 (100)					

PPV: Positive predictive value; NPV: Negative predictive value.

## Data Availability

The data can be obtained from the relevant author upon request.
